# Foveal Protection with Viscoelastic Material during Removal of Posterior Segment Foreign Bodies

**Published:** 2010-01

**Authors:** Touka Banaee, Maria Sharepoor

**Affiliations:** Eye Research Center and Khatam-al-Anbia Hospital, Mashhad University of Medical Sciences, Mashhad, Iran

**Keywords:** Intraocular Foreign Body, Pars Plana Vitrectomy, Viscoelastic Materials, Ophthalmic Viscosurgical Devices

## Abstract

Foreign bodies may drop during removal from the posterior segment and result in foveal damage. Due to high specific gravity and viscosity, ophthalmic viscosurgical devices (OVDs) can dampen and redirect the force of the dropping foreign body and therefore protect the fovea. Herein we describe our technique of foveal protection with OVDs and briefly demonstrate the results in five eyes with large posterior segment foreign bodies.

## INTRODUCTION

Removal of posterior segment intraocular foreign bodies (IOFBs) is usually performed after pars plana vitrectomy. This may be accomplished utilizing various methods and is technically demanding. Use of intraocular magnets has simplified removal of magnetic foreign bodies from the retinal surface, but for extraction through the sclerotomy, a firmer grasp with intraocular forceps is required. Non-magnetic foreign bodies must be removed with intraocular baskets or forceps.

The procedure incurs risks of foreign body drop, iatrogenic retinal breaks, intraocular hemorrhage and retinal detachment. Drop of the IOFB during removal may cause retinal damage and can be catastrophic if it strikes the unaffected fovea. This can occur when the IOFB is grasped with forceps in the vitreous cavity or at the time of extraction through the sclerotomy. This complication is uncommon during removal of small IOFBs because most of them can be removed with intraocular baskets. Even when removed with forceps, only slight grasping force can overcome the weight of the IOFB. A large and heavy IOFB with irregular surface is most prone to drop during grasping. The fluid filled vitreous cavity can somewhat decrease the weight of the IOFB according to Archimedes’ law and dampen acceleration of the foreign body, but this still seems inadequate to prevent retinal damage in case of large heavy particles. To solve this problem, instillation of perfluorocarbon liquids (PFCLs) on the retina before removal of the foreign body has been advocated.^1^,^2^ The specific gravity of PFCLs is 1.89–2.03 times greater than that of water,^3^ therefore they remain on the retina, but the difference in specific gravity between PFCLs and water is not large enough to affect the weight of IOFBs especially those composed of metal or stone. Moreover, the viscosities of PFCLs are approximately equal to that of water (*i.e.* 0.8 to 8.03 cSt)^4^ and there is no added resistance against the dropping foreign body. Therefore a high viscosity material with specific gravity greater than water would be ideal for this purpose. Of commercially available materials for intraocular use, ophthalmic viscosurgical devices (OVDs) seem to possess both of the mentioned characteristics. There are two types of OVDs: dispersive and cohesive. Dispersive viscoelastics (such as Viscoat® and Vitax®) have low molecular weight, low surface tension and high viscosity, whereas cohesive ones (such as Healon® and Amvisc®) have high molecular weight, high surface tension, and high pseudoplasticity.^5^

Dispersive viscoelastics exert resistance against the dropping foreign body because of their high viscosity and dampen the kinetic energy of the IOFB, reducing the risk of damage in case of drop on the fovea. When the dropping foreign body comes in contact with the viscoelastic material, it is slowed down and may change direction. Theoretically, cohesive materials are better than dispersive ones for this purpose; their greater surface tension provides additional resistance against the force of the drop and may even prevent the IOFB from entering the OVD bubble causing it to float over the bubble. But the major concern with their use is difficulty in removing them from the posterior segment through a 20-gauge needle. Another concern is that by forming a bubble with convex surface, the IOFB may float and displace peripherally where removal may be more difficult and entail complications.

## SURGICAL TECHNIQUE

An important step during surgery for IOFBs is complete removal of the vitreous gel after induction of posterior vitreous detachment (PVD), before attempting to remove the foreign body. Although cortical vitreous is a very good buffer and offers a protecting shell for the retina if a drop should occur, presence of vitreous during IOFB removal increases the risk of tractional retinal break formation. This can occur because of vitreous incarceration into the enlarged sclerotomy or during introduction and extraction of large foreign body forceps. Use of triamcinolone during vitrectomy helps visualization of clear vitreous to ensure adequate vitrectomy before proceeding to other steps of the surgery.

Viscoelastic material must be injected before manipulation of the foreign body. About half to one-third of the vitreous cavity should be filled with OVD before IOFB removal is attempted. After removal of the foreign body, thorough extraction of the viscoelastic material is indicated to prevent excessive postoperative inflammation.^6^ Removal of the viscoelastic material can be accomplished passively or actively.

We have used this technique for removal of all large IOFBs since 2006. Five cases with large IOFBs were operated since then. Preoperative characteristics of the patients and the foreign bodies are presented in Table 1. We filled about one-third of the vitreous cavity with dispersive viscoelastic material (Coatel, Bausch & Lomb, Waterford, Ireland) prior to IOFB removal in 5 eyes with large IOFBs. Coatel is a sterile, isotonic, non-pyrogenic viscoelastic solution composed of highly purified 2% hydroxypropyl methylcellulose with molecular weight greater than 80,000 Daltons, specific gravity greater than water, osmolarity of 285±32 mOsm and viscosity of 4,000±1,500 cSt.^5^ Three cases (cases 1, 2, and 4) of IOFB drop occurred during removal. The viscoelastic material effectively protected the fovea and caused redirection of the vector of the IOFB drop to more peripheral parts of the fundus. There were no visible changes in the fovea intraoperatively. Two patients (cases 1 and 2) enjoyed good visual acuity with normal fundus postoperatively. Case #4 experienced proliferative vitreoretinopathy and redetachment of the peripheral retina and underwent two additional pars plana vitrectomies. His vision was limited because of tractional macular distortion. None of the other 4 cases developed epiretinal membranes.

One important observation during surgery was that the presence of viscoelastic material facilitated removal of IOFBs from the retinal surface. One of the forceps blades was used to slightly elevate the IOFB from the retinal surface, a thin layer of viscoelastic tracked beneath the foreign body preventing it from re-approximation to the retina in cases which the foreign body could not be grasped with the first attempt.

## CONCLUSION

Use of viscoelastic materials in the vitreous cavity during removal of large and heavy IOFBs adds to the safety of surgery and can protect the fovea in cases when inadvertent drops occur. Removal of dispersive OVDs from the vitreous cavity requires only a few minutes additional time and can be easily accomplished with a flute needle. It is prudent to completely remove all viscoelastic material from the vitreous cavity to avoid inflammation.

## Figures and Tables

**Table 1:** Pre- and postoperative characteristics of the patients

Cases	1	2	3	4	5
Sex /Age	M/64	M/20	M/31	M/24	M/27
Primary VA	5/200	20/50	20/20	20/20	20/20
Preoperative complications	Cataract	Cataract	Cataract	Endophthalmitis & RRD	RRD, Cataract
Entrance site	Sclera	Cornea	Cornea	Sclera	Cornea
IOFB location	Intravitreal	Embedded in retina	Embedded in retina	Embedded in retina	Intravitreal
Size of IOFB (mm)	11*3*2	10*3*2	3*5*7	3*2.5*0.5	1.5*3*2.75
IOFB removal technique	Magnet & forceps	Forceps	Forceps	Forceps	Forceps
Incision used for removal	Limbus	Widened sclerotomy	Corneal stab incision	Widened sclerotomy	Widened sclerotomy
Surgical procedures at the time of FB removal	Lensectomy, ELP	SB (240) cryotherapy posterior to widened sclerotomy	Phacoemulsification+IOL, ELP	SB (240), ELP, Cryotherapy	Cryotherapy, PPV, C2F6
Post FB removal surgical procedures	Secondary IOL	-	Intravitreal gas + cryotherapy due to RRD	1- PPV+Retinotomy+SO (1300) 2- Lenx+re PPV +SO (5700)	Phacoemulsification+IOL
Postoperative VA	20/30	20/20	20/30	20/200	20/70

M, male; VA, visual acuity; RRD, rhegmatogenous retinal detachment; IOFB, intraocular foreign body; SB, scleral buckling; ELP, endolaser photocoagulation; IOL, intraocular lens; PPV, pars plana vitrectomy; Lenx, lensectomy; SO, silicone oil.
